# Correlation-Based Multiplexing of Complex Amplitude Data Pages in a Holographic Storage System Using Digital Holographic Techniques

**DOI:** 10.3390/polym9080375

**Published:** 2017-08-18

**Authors:** Teruyoshi Nobukawa, Takanori Nomura

**Affiliations:** 1 Graduate School of Systems Engineering, Wakayama University, 930 Sakaedani, Wakayama 640-8510, Japan; 2 Faculty of Systems Engineering, Wakayama University, 930 Sakaedani, Wakayama 640-8510, Japan; nom@sys.wakayama-u.ac.jp

**Keywords:** holography, holographic data storage, phase hologram, digital holography, photopolymer

## Abstract

Holographic recording media can store the amplitude and the phase, or the complex amplitude, of a beam on the basis of holography. Owing to this characteristic, digital data can be encoded onto the complex amplitude of a signal beam in holographic data storage. However, most of conventional holographic storage systems encode digital data onto the amplitude alone because there are difficulties for modulating and detecting the phase. To solve the difficulties, a holographic storage system using digital holographic techniques has been proposed. With the help of digital holographic techniques, it is possible to modulate and detect the complex amplitude of a signal beam. Moreover, the proposed system can modulate the complex amplitude of a reference beam. In this paper, by making use of the capability, a correlation-based multiplexing with uncorrelated reference beams is demonstrated in the proposed system. Multiple holograms can be recorded in the same volume of a recording medium with no need for mechanical movements. Experimental results show that the proposed system with a correlation-based multiplexing can improve the storage capacity and can utilize the full potential of a recording medium without crosstalk noise stem from the optical setup.

## 1. Introduction

Holographic data storage has been considered to be an attractive candidate for the next-generation optical data storage technology because of its potential for simultaneously achieving huge capacity and fast transfer rates [1,2]. In holographic data storage, digital data to be stored are converted into a two-dimensional data array called a data page. According to the data page, the amplitude distribution of a beam is spatially modulated using a spatial light modulator (SLM). The modulated beam, called a signal beam, interferes with a reference beam in a recording medium. The three-dimensional intensity distribution of the interference pattern is recorded throughout the volume of a recording medium via modulation of the real and/or imaginary part of the dielectric constant. As a result, digital data are stored as a volume hologram in a recording medium. To retrieve the digital data, the volume hologram is illuminated with the reference beam that is used during recording. The portion of the reference beam is diffracted by the volume hologram. The diffracted beam is a replica of the signal beam. By detecting the intensity distribution of the diffracted beam using an image sensor, the recorded digital data can be readout. When recording subsequent volume holograms of data pages, the optical property of a reference beam and/or the three-dimensional position of a recording medium are changed. A lot of holograms can therefore be recorded in the same volume of a recording medium. This recording process is known as multiplexing. In order to improve storage capacity and develop a commercial product, various multiplexing methods such as angle, shift, phase-code, and peristrophic multiplexing methods have been proposed and demonstrated [1,3]. In addition to the multiplexing methods, several recording medium [4,5,6,7], optical systems [8,9,10,11,12,13], recording techniques [14,15,16], and retrieving techniques [17,18,19] have been developed. Through the progress in supporting technologies, the recording density of more than 1 Tbit/in${}^{2}$ has been experimentally archived [20,21].

In general, holographic recording media are capable of recording and reconstructing the amplitude and the phase of a beam on the basis of holography. In addition to the amplitude, the phase should therefore be used as an information carrier in holographic data storage. By encoding digital data onto the amplitude and the phase, or the complex amplitude, it is possible to increase the amount of information in a single data page. We refer to such a data page as a complex amplitude data page. Recording and retrieving complex amplitude data pages result in large storage capacity and fast transfer rates compared with traditional holographic storage systems that encode digital data onto the amplitude alone. However, most conventional holographic storage systems, including the abovementioned systems, cannot utilize the phase as an information carrier. The current SLMs modulate either the amplitude of the phase, and thus it is impossible to modulate the complex amplitude of a beam using a single SLM on the pixel-by-pixel basis. Moreover, image sensors are insensitive to the phase of a beam. Traditional holographic storage systems therefore cannot utilize the full potential of a recording medium. The introduction of two SLMs and an interferometer enables an optical setup to modulate and detect the complex amplitude distribution. However, this makes an optical setup large, complicated, and expensive. Due to the abovementioned difficulties, few experimental studies have reported on recording complex amplitude data pages although the significance of the phase has been recognized [22,23,24].

For the purpose of recording complex amplitude data pages in a simple optical setup, we have proposed a holographic storage system using digital holography [25]. Digital holography is the technology that records and reconstructs a hologram with electric devices and signal processing, unlike analog holography. Owing to digital holography, the proposed system can modulate and detect the complex amplitude distribution of a signal beam in a common-path setup consists of a single SLM and a single image sensor. In a preliminary experiment, we have verified that complex amplitude data pages can be recorded and retrieved with shift multiplexing [25]. In shift multiplexing, multiple volume holograms are partially overlapped and recorded by shifting a recording medium. Reducing the shift distance of a recording medium results in large storage capacity. However, the shift distance should be at least several micrometers to suppress crosstalk noise from adjacent volume holograms [26]. The crosstalk noise in shift multiplexing is mainly caused by a recording system rather than a recording medium. If the crosstalk noise is suppressed without shifting a recording medium in the proposed system, it is possible to utilize the full potential of a recording medium and improve the storage capacity of holographic data storage.

In this paper, we apply a correlation-based multiplexing to the proposed system in order to suppress the crosstalk noise without shifting a recording medium. In the proposed system, it is possible to manipulate the complex amplitude distribution of a reference beam in addition to a signal beam. Owing to this capability, the proposed system therefore has a possibility of implementing a correlation-based multiplexing using uncorrelated reference beams instead of shift multiplexing. In the correlation-based multiplexing, also called phase-code multiplexing, multiple data pages are recorded using uncorrelated reference beams without displacing a recording medium. A straightforward way for generating uncorrelated reference beams is the use of multiple random phase masks provided by an SLM or a diffuser [27,28,29]. The use of random phase masks, however, wastes the light energy of a reference beam because the spatial-frequency spectrum of a reference beam is widely spread and filtered with an aperture. Instead of random phase masks, we make use of computer-generated reference patterns (CGRPs) [30,31]. By using CGRPs, it is possible to control the spectrum distribution of a reference beam, and thus the light efficiency can be improved.

This paper is structured as follows. In Section 2, we describe our proposed holographic storage system with digital holographic techniques and a correlation-based multiplexing method using uncorrelated CGRPs. In Section 3, we experimentally demonstrate correlation-based multiplexing of complex amplitude data pages using uncorrelated CGRPs. Finally, a conclusion is given in Section 4.

## 2. Correlation-Based Multiplexing in a Holographic Storage System with Digital Holographic Techniques

**Figure 1:**
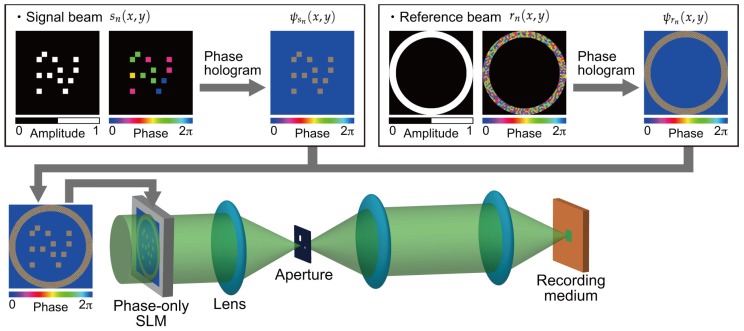
Recording process in a holographic storage system using digital holographic techniques.

Figure 1 shows a schematic of a recording process in a holographic storage system using digital holographic techniques. This optical setup is based on a collinear holographic storage system where signal and reference beams propagate along a common optical axis [8,32,33]. The conventional collinear holographic storage system is not capable of modulating and detecting the complex amplitude distribution of a beam. Unlike the conventional collinear holographic storage system, our proposed system can modulate and detect the complex amplitude distribution of a beam [25]. The principle of a correlation-based multiplexing is related to an optical correlator using a volume hologram [34,35]. Recording and retrieving processes of complex amplitude data pages with uncorrelated as described below.

Let $s_{n}\left(x,y\right)$ and $r_{n}\left(x,y\right)$ denote a *n*th signal beam contains a *n*th complex amplitude data page to be stored and *n*th reference beam, respectively. By a correlation-based multiplexing, *N* signal beams, $s_{1}$, ⋯, $s_{N}$ are recorded in the same volume of a recording medium with *N* uncorrelated reference beams, $r_{1}$, ⋯, $r_{N}$. Note that the reference beams, $r_{1}$, ⋯, $r_{N}$, are uncorrelated with each other. Signal and reference beams are expressed as

(1) $$\begin{array}{ccc} s_{n}\left(x,y\right) & = & a_{s_{n}}\left(x,y\right)\exp\left\{i\phi_{s_{n}}\left(x,y\right)\right\}, \end{array}$$

(2) $$\begin{array}{ccc} r_{n}\left(x,y\right) & = & a_{r}\left(x,y\right)\exp\left\{i\phi_{r_{n}}\left(x,y\right)\right\}, \end{array}$$

respectively. $a_{s_{n}}$ and $\phi_{s_{n}}$ denote the amplitude and the phase of a signal beam. Similarly, $a_{r}$ and $\phi_{r_{n}}$ denote the amplitude and the phase of a reference beam. Note that the amplitude of a reference beam, $a_{r}\left(x,y\right)$, is the same regardless of *n*. By solely changing the phase $\phi_{r_{n}}$, it is possible to generate uncorrelated reference beams $r_{n}\left(x,y\right)$ [31].

During the recording process, phase holograms [36,37,38,39], $\psi_{s_{n}}\left(x,y\right)$ and $\psi_{r_{n}}\left(x,y\right)$, for generating signal and reference beams are designed in a computer on the basis of following equations:

(3) $$\begin{array}{ccc} \psi_{s_{n}}\left(x,y\right) & = & a_{s_{n}}\left(x,y\right)\left\{\phi_{s_{n}}\left(x,y\right)+\phi_{c}\left(x,y\right)\right\}, \end{array}$$

(4) $$\begin{array}{ccc} \psi_{r_{n}}\left(x,y\right) & = & a_{r}\left(x,y\right)\left\{\phi_{r_{n}}\left(x,y\right)+\phi_{c}\left(x,y\right)\right\},\psi_{r_{m}}\left(x,y\right), \end{array}$$

respectively. $\phi_{c}\left(x,y\right)$ is linear phase distribution,

(5) $$\begin{array}{c} \phi_{c}\left(x,y\right)=2\pi \left(f_{x}x+f_{y}y\right), \end{array}$$

where $f_{x}$ and $f_{y}$ are spatial carrier frequencies. $\phi_{c}\left(x,y\right)$ is required to separate a desired component from the spatial frequency spectra in a Fourier plane. It should be noted that Equations (3) and (4) are valid when the amplitude distribution consists of ON (bright) and OFF (dark) cells [39]. In the case of encoding digital data onto the gray-scale level of the amplitude [40], it is necessary to modify the equations. The phase holograms, $\psi_{s_{n}}\left(x,y\right)$ and $\psi_{r_{n}}\left(x,y\right)$, are superimposed and displayed on a phase-only SLM. The phase-only SLM modulates the phase distribution of a plane wave. The modulated beam, $\exp\{i\psi_{s_{n}}\left(x,y\right)\}$ and $\exp\{i\psi_{r_{n}}\left(x,y\right)\}$, are optically two-dimensional Fourier transformed by a lens. An aperture removes undesired spectra from the phase-modulated beams. The filtered beam is optically Fourier transformed again. As a result, the desired signal and reference beams, $s_{n}$ and $r_{n}$, can be obtained on the conjugate plane of the phase-only SLM. Although the liner phase $\phi_{c}\left(x,y\right)$ is superimposed on the signal and reference beams, it is not significant because the linear phase is used as a spatial frequency carrier during retrieving process. The principle of the abovementioned phase hologram technique is described in [36,37,38,39]. The generated signal and reference beams are optically Fourier transformed to form an interference pattern in a recording medium. The resulting interference pattern is

(6) $$\begin{array}{ccc} H_{n}\left(u,v\right) & = & |R_{n}\left(u-f_{x},v-f_{y}\right)+S_{n}\left(u-f_{x},v-f_{y}\right){|}^{2}\\ & = & |R_{n}\left(u-f_{x},v-f_{y}\right){|}^{2}+{\left|S_{n}\left(u-f_{x},v-f_{y}\right)\right|}^{2}\\ & & +R_{n}^{*}\left(u-f_{x},v-f_{y}\right)S_{n}\left(u-f_{x},v-f_{y}\right)\\ & & +R_{n}\left(u-f_{x},v-f_{y}\right)S_{n}^{*}\left(u-f_{x},v-f_{y}\right), \end{array}$$

where $R_{n}\left(u,v\right)$ and $S_{n}\left(u,v\right)$ are the Fourier spectra of reference and signal beams, respectively. Note that the spectra of the signal and reference beams are spatially shifted from the optical axis due to the liner phase in Equation (5). A dielectric constant of a recording medium is changed depending on the intensity of the interference pattern. By illuminating the interference patterns of all signal beams $s_{1}\left(x,y\right)$, ⋯, $s_{N}\left(x,y\right)$ without shifting a recording medium, the transmittance of a recoding medium is simply given by the summation of their interference patterns,

(7) $$\begin{array}{c} H\left(u,v\right)=C\sum_{n=1}^{N}H_{n}\left(u,v\right), \end{array}$$

where *C* is constant determined by a recording medium and recording conditions. Although the equation describes the transmittance change induced by the intensity hologram, it is valid for the case where the interference pattern is recorded as a small refractive index change. As a result, a volume hologram of signal beams can be recorded in a recording medium. Note that the effect of a thickness of a recording medium is ignored in this paper. This is valid to describe the behavior of correlation-based multiplexing.

**Figure 2:**
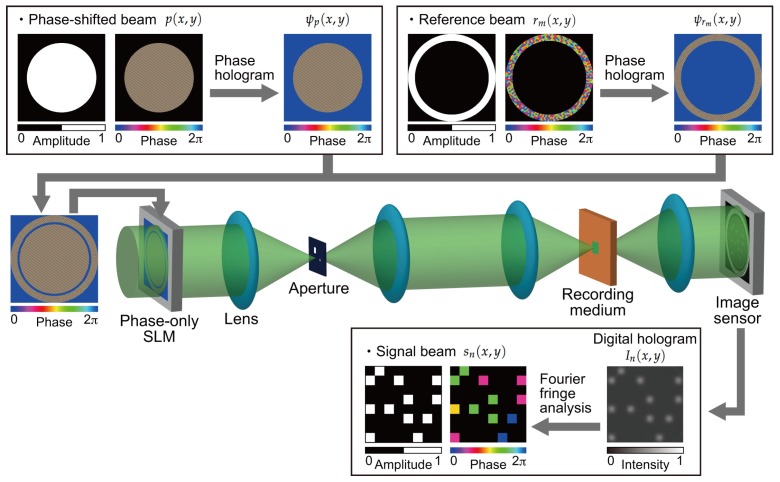
Retrieving process in a holographic storage system using digital holographic techniques.

Figure 2 shows a schematic of a retrieving process in a holographic storage system using digital holographic techniques. During the retrieving process, a reference beam used for retrieving $r_{m}\left(x,y\right)$ (1≤m≤N) is generated from a phase-only SLM in the same way as when a volume hologram is recorded. At the same time, an addition beam is generated for the detection of the complex amplitude distribution of a reconstructed signal beam. We refer to this additional beam as a phase-shifted beam, and this beam is given by

(8) $$\begin{array}{ccc} p(x,y) & = & a_{p}\left(x,y\right)\exp\left\{i\phi_{p}\left(x,y\right)\right\}, \end{array}$$

where $a_{p}$ and $\phi_{p}$ denote the amplitude and the phase, respectively. $\phi_{p}$ is linear phase distribution:

(9) $$\begin{array}{c} \phi_{p}\left(x,y\right)=2\pi \left(f_{x_{p}}x+f_{y_{p}}y\right), \end{array}$$

where $f_{x_{p}}$ and $f_{y_{p}}$ are spatial carrier frequencies. For the generation of the phase-shifted beam, a phase hologram

(10) $$\begin{array}{ccc} \psi_{p}\left(x,y\right) & = & a_{p}\left(x,y\right)\phi_{p}\left(x,y\right) \end{array}$$

is superimposed onto the phase hologram of a reference beam $\psi_{r_{m}}\left(x,y\right)$. The superimposed phase pattern is displayed on a phase-only SLM. According to the phase holograms, the phase distribution of a plane wave is modulated. The modulated beam is spatially band-pass filtered with an aperture, and the reference and phase-shifted beams can be obtained on the conjugate plane of the phase-only SLM. The reference beam is optically Fourier transformed by a lens and incident on the recording medium, resulting in a diffracted beam,

(11) $$\begin{array}{ccc} D_{m}\left(u,v\right) & = & C\sum_{n=1}^{N}\left(\right|R_{n}\left(u-f_{x},v-f_{y}\right){|}^{2}+|S_{n}\left(u-f_{x},v-f_{y}\right){|}^{2})R_{m}\left(u-f_{x},v-f_{y}\right)\\ & & +C\sum_{n=1}^{N}S_{n}{\left(u-f_{x},v-f_{y}\right)}^{*}R_{n}\left(u-f_{x},v-f_{y}\right)R_{m}\left(u-f_{x},v-f_{y}\right)\\ & & +C\sum_{n=1}^{N}S_{n}\left(u-f_{x},v-f_{y}\right)R_{n}^{*}\left(u-f_{x},v-f_{y}\right)R_{m}\left(u-f_{x},v-f_{y}\right). \end{array}$$

The first term is the zeroth order beam, which can be removed by a spatial filtering. The second term is widely spread out on an image sensor owing to the convolution between $r_{n}$ and $r_{m}$. In addition, its intensity is sufficiently small owing to the characteristic of a volume hologram. The third term gives the reconstructed beam of interest. The third term of the diffracted beam is Fourier transformed by a lens, and the complex amplitude on an image sensor is given by,

(12) $$\begin{array}{ccc} d_{m}\left(x,y\right) & = & C\exp\left\{i2\pi \left(f_{x}x+f_{y}y\right)\right\}\sum_{n=1}^{N}s_{n}\left(x,y\right)⊗r_{n}\left(x,y\right)⊕r_{m}\left(x,y\right), \end{array}$$

where ⊗ and ⊕ denote convolution and correlation operators, respectively. When n=m, the auto correlation, $r_{n}\left(x,y\right)⊕r_{n}\left(x,y\right)$, closely approximate a delta function, and a signal beam $s_{n}\left(x,y\right)$ can be obtained. Because the reference beams are uncorrelated with each other, the crosstalk noise due to a cross correlation $r_{n}\left(x,y\right)⊕r_{m}\left(x,y\right)$ (n≠m) is sufficiently small.

The complex amplitude distribution of the reconstructed signal beam is detected using a Fourier fringe analysis [41] as follows. On an image sensor, the reconstructed signal beam without crosstalk noise interferes with the phase-shifted beam that propagates from the conjugate plane of the phase-only SLM. Within the area of interest on an image sensor, the interference pattern

(13) $$\begin{array}{ccc} I_{n}\left(x,y\right) & = & |Cs_{n}\left(x,y\right)⊗r_{n}\left(x,y\right)⊕r_{n}\left(x,y\right)\exp\left\{i2\pi \left(f_{x}x+f_{y}y\right)\right\}\\ & & +a_{p}\left(x,y\right)\exp\left\{i2\pi \left(f_{x_{p}}x+f_{y_{p}}y\right)\right\}{|}^{2} \end{array}$$

is captured as a digital hologram. Setting C=1 and $a_{p}\left(x,y\right)=1$, and regarding $r_{n}\left(x,y\right)⊕r_{n}\left(x,y\right)$ as a delta function for simplicity, Equation (13) becomes

(14) $$\begin{array}{ccc} I_{n}\left(x,y\right) & = & |s_{n}\left(x,y\right)\exp\left\{i2\pi \left(f_{x}x+f_{y}y\right)\right\}+\exp\left\{i2\pi \left(f_{x_{p}}x+f_{y_{p}}y\right)\right\}{|}^{2}\\ & = & |s_{n}{\left(x,y\right)|}^{2}+1\\ & & +s_{n}\exp\left\{i2\pi \left(f_{xs}x+f_{ys}y\right)\right\}\\ & & +s_{n}^{*}\left(x,y\right)\exp\left\{i2\pi \left(-f_{xs}x-f_{ys}y\right)\right\}, \end{array}$$

where $f_{xs}$ and $f_{ys}$ are given by

(15) $$\begin{array}{ccc} f_{xs} & = & f_{x}-f_{x_{p}}, \end{array}$$

(16) $$\begin{array}{ccc} f_{ys} & = & f_{y}-f_{y_{p}}, \end{array}$$

respectively. The captured digital hologram is digitally Fourier transformed using a computer:

(17) $$\begin{array}{ccc} F[I_{n}\left(x,y\right)] & = & F[|s_{n}\left(x,y\right){|}^{2}+1]+S_{n}\left(u-f_{xs},v-f_{ys}\right)+S_{n}^{*}\left(-u-f_{xs},-v-f_{ys}\right), \end{array}$$

where F[X] denotes the Fourier transform operator. The first term is the zeroth order component. The second term is the Fourier spectrum of a signal beam. The third term is the complex conjugate of the second term. Because each component is separately placed in the Fourier plane, it is possible to extract only $S_{n}\left(u-f_{xs},v-f_{ys}\right)$. By removing known frequencies, $f_{xs}$ and $f_{ys}$, and digitally inverse Fourier transforming, the complex amplitude data page can be detected. The original data page $s_{n}\left(x,y\right)$ can therefore be retrieved. Through the above procedure using other reference beams, individual complex amplitude data pages can be independently retrieved from the multiplexed volume holograms.

## 3. Experimental Demonstration

In this section, we experimentally demonstrate holographic data recording of complex amplitude data pages to verify the feasibility of the correlation-based multiplexing in the proposed system. First, we record a single data page using a single CGRP to show that the proposed system can utilize the complex amplitude as an information carrier. Subsequently, we investigate the selectivity of recording holograms using two uncorrelated CGRPs. Finally, we implement the correlation-based multiplexing using two uncorrelated CGRPs.

### 3.1. Single Recording

**Figure 3:**
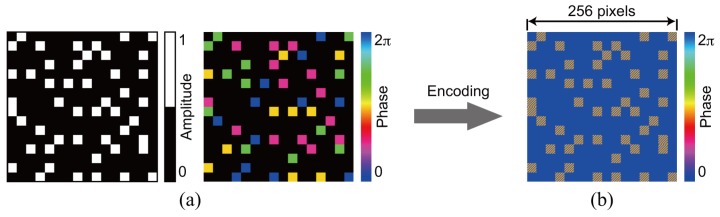
Data page 1. (**a**) complex amplitude distribution and (**b**) phase hologram.

**Figure 4:**
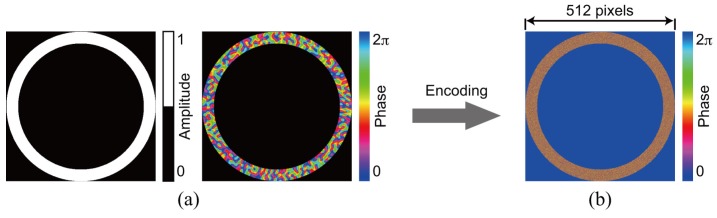
Reference beam for recording data page 1. (**a**) complex amplitude distribution and (**b**) phase hologram.

**Figure 5:**
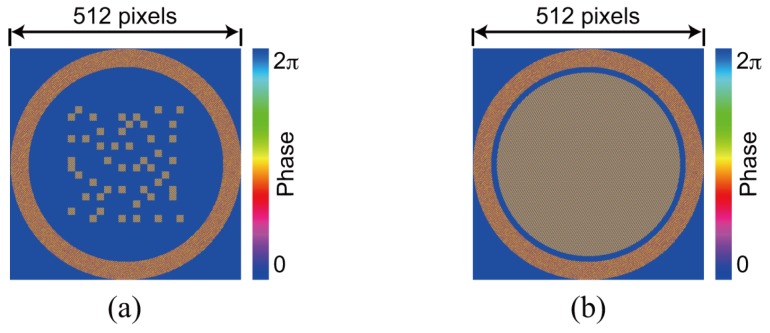
Superimposed phase holograms for (**a**) recording and (**b**) retrieving the data page 1.

**Figure 6:**
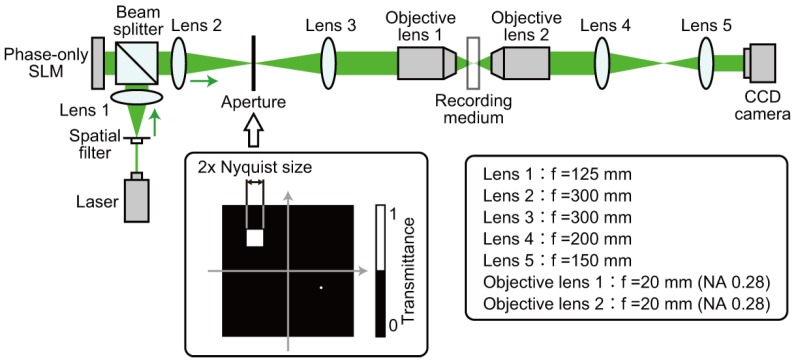
Experimental setup for holographic data recording.

**Figure 7:**
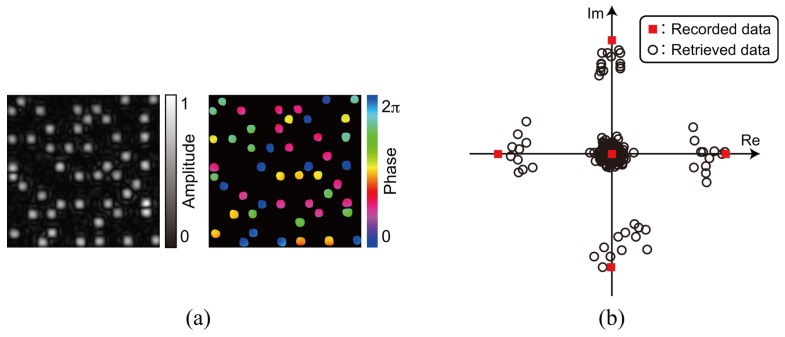
Retrieved complex amplitude data page 1. (**a**) complex amplitude distribution; (**b**) averaged complex amplitude values on a complex plane.

For the demonstration of single data recording, we recorded and retrieved a complex amplitude data page 1 depicted in Figure 3a. To generate the amplitude distribution shown in Figure 3a, we used a 3:16 coding. In this coding, a single symbol consists of three ON and thirteen OFF cells in 4 × 4 cells [42]. The coding method can suppress interpixel crosstalk noise [43,44]. The phase distribution consists of four-level phase values, 0, π/2, π, and 3π/2. Note that the phase distribution where the amplitude value is 0 is represented by the black in Figure 3a. For the generation of the signal beam that possesses the complex amplitude data page 1, a phase hologram is designed on the basis of Equation (3). Figure 3b shows a phase hologram for generating a signal beam. Figure 4a shows the complex amplitude distribution of a reference beam that is used for recoding a volume hologram of the signal beam. The phase distribution, the CGRP, can be designed by a simulated annealing method [30]. According to the complex amplitude distribution, a phase hologram shown in Figure 4b is obtained. The phase holograms shown in Figure 3b and Figure 4b are superimposed, which results in the phase hologram shown in Figure 5a. The phase hologram was displayed on the phase-only SLM in the optical setup shown in Figure 6. In the optical setup, a laser diode with a wavelength of 532 nm was used as a coherent light source. A plane wave was obtained though a spatial filter and a collimator lens. The plane wave was incident on a phase-only SLM (X10468-01, Hamamatsu Photonics K. K., Shizuoka, Japan) with 800 × 600 pixels and a pixel pitch of 20 μm. The phase-modulated beam was band-pass filtered with a square aperture to generate signal and reference beams and to restrict a recording area. The width of the square aperture is the 2× Nyquist size [1,3]. By the band-pass filtering, signal and reference beams were generated on the conjugate plane of the phase-only SLM. These beams were Fourier transformed, and the interfere pattern was recorded in a photopolymer material (Kyoeisha Chemical Co., Osaka, Japan) with a thickness of 400 μm. During the retrieving process, the phase hologram shown in Figure 5b was displayed on the phase-only SLM. In the conjugate plane of the phase-only SLM, reference and phase-shifted beams were generated. The volume hologram was illuminated with the reference beam, and thus the signal beam was reconstructed. The reconstructed signal beam interfered with the phase-shifted beam on a charge coupled device (CCD) camera with 1280 × 960 pixels and a pixel pitch of 4.65 μm. By applying the Fourier fringe analysis, the complex amplitude distribution of the signal beam can be detected. Figure 7a shows the complex amplitude distribution of the reconstructed signal beam. Note that in the phase distribution of Figure 7a, the phase values where the amplitude values are less than 0.3 are represented by the black. The detected complex amplitude is decoded as follows [25]. The complex amplitude distribution was divided to 16 × 16 areas that are the same as the number of cells in a single data page. In each area, the complex amplitude values were averaged. The averaged complex amplitude values were plotted on a complex plane, as shown in Figure 7b. In Figure 7b, the solid squares denote the complex amplitude values of the recorded data. The open circles denote the average complex amplitude values of retrieved data. The amplitude of retrieved data are normalized by that of recorded data in Figure 7b. Each retrieved data are regarded as the nearest-neighbor recorded data on the complex plane, and thereby the retrieved data can be decoded. By applying the above decoding process, the original data page was successfully retrieved from the complex amplitude distribution shown in Figure 7a. From the above experiment, we have verified that the proposed system can record and retrieve a single complex amplitude data page. In the next subsection, we demonstrate the correlation-based multiplexing.

### 3.2. Correlation-Based Multiplexing

**Figure 8:**
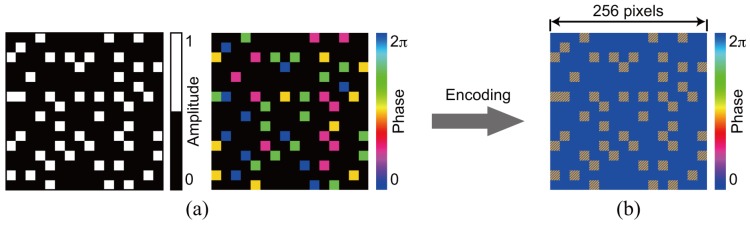
Data page 2. (**a**) complex amplitude distribution and (**b**) phase hologram.

**Figure 9:**
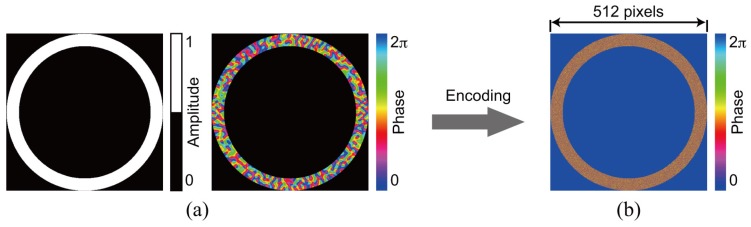
Reference beam for recording data page 2. (**a**) complex amplitude distribution and (**b**) phase hologram.

**Figure 10:**
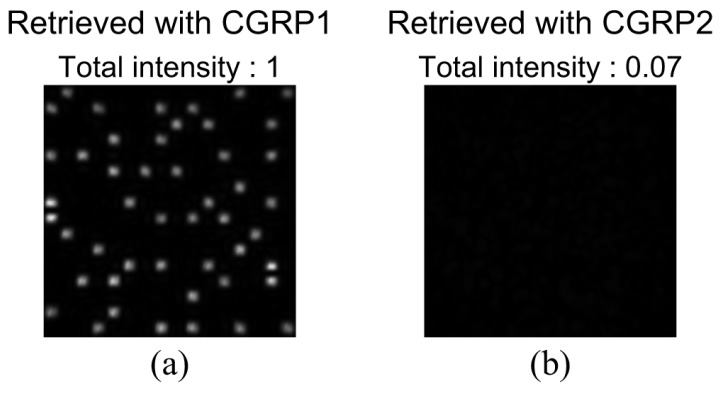
Intensity distributions of a reconstructed beam from a volume hologram of the data page 1 by illuminating with (**a**) CGRP1 and (**b**) CGRP2.

**Figure 11:**
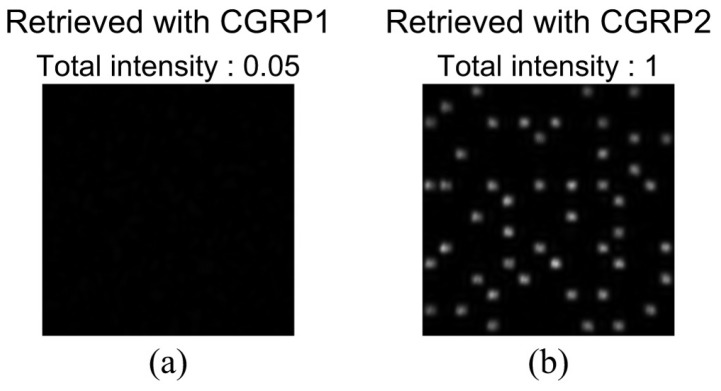
Intensity distributions of a reconstructed beam from a volume hologram of the data page 2 by illuminating with (**a**) CGRP1 and (**b**) CGRP2.

To show the feasibility of the correlation-based multiplexing, we record two complex amplitude data pages shown in Figure 3 and Figure 8. Each data page is recorded and retrieved using two CGRPs shown in Figure 4 and Figure 9, respectively. The CGRP2 can be designed using an uncorrelated random phase mask as an initial state in a simulated annealing method [31]. The maximum intensity of the cross correlation between the CGRP1 and the CGRP2 is 0.005, which is normalized by that of the auto correlation of CGRPs. Before implementing the correlation-based multiplexing, we first investigate the selectivity of the two uncorrelated CGRPs. The complex amplitude data page 1 was recorded using the CGRP1 in the same way as the process described in Section 3.1. By illuminating the volume hologram with the CGRP1, the intensity distribution was obtained, as shown in Figure 10a. Note that we did not detect the complex amplitude distribution using the phase-shifted beam because only the intensity of the reconstructed beam is of interest to investigate the selectivity. When the volume hologram was illuminated with the CGRP2, the data page 1 cannot be reconstructed as shown in Figure 10b. The total intensity of the reconstructed signal beam of Figure 10b is 7% of that of Figure 10a. Next, we recorded the complex amplitude data page 2 using the CGRP2 in the different position of a recording medium where the data page 1 is recorded. Figure 11a,b show the intensity distributions of reconstructed signal beams using the CGRP1 and CGRP2, respectively. In contrast to Figure 10, the data page 2 can be retrieved when the reference beam with the CGRP2 is illuminated. The total intensity of the reconstructed signal beam in Figure 11a is 5% of that of Figure 11b. From the above experiments, we experimentally verified that the selectivity of uncorrelated CGRPs.

**Figure 12:**
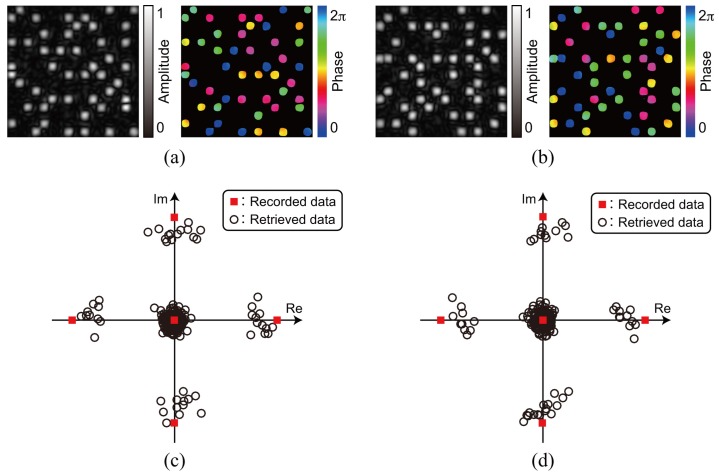
Experimental results of correlation-based multiplexing. Complex amplitude distribution of the retrieved (**a**) data page 1 and (**b**) data page 2. Averaged complex amplitude values of the retrieved (**c**) data page 1 and (**d**) data page 2 on a complex plane.

Finally, we demonstrate the correlation-based multiplexing of two data pages using two uncorrelated CGRPs. The volume hologram of the complex amplitude data 1 was recorded using the CGRP1. After that, the volume hologram of the complex amplitude data page 2 was recorded using the CGRP2 in the same volume of a recording medium without shifting a recording medium. The recording medium contains two volume holograms was sequentially illuminated with the reference beam with the CGRP1 and the CGRP2. Figure 12a,b show the complex amplitude distribution of the reconstructed signal beams by using the CGRP1 and the CGRP2, respectively. There is sufficiently small crosstalk noise owing to the uncorrelated CGRPs. Each of the complex amplitude distributions is averaged and plotted on a complex plane, as shown in Figure 12c,d. By the decoding process, the original data pages can be successfully retrieved without any error. The experimental results show that the proposed system using the correlation-based multiplexing can record and retrieve complex amplitude data pages without shifting a recording medium.

## 4. Conclusions

We have demonstrated the correlation multiplexing of complex amplitude data pages in our proposed holographic storage system with digital holographic techniques. Our proposed system can utilize the complex amplitude distribution as an information carrier in a simple and compact optical setup, which allows multilevel recording. In addition, it is possible to modulate the complex amplitude distribution of a reference beam. By making use of the capability, it is possible to implement the correlation-based multiplexing using uncorrelated reference beams. With the correlation-based multiplexing, multiple volume holograms of complex amplitude data pages can be recorded in the same volume of a recording medium without shifting a recording medium. This allows stable multiplexing, which is useful for realizing a practical system. Experimental results show that the proposed system with the correlation multiplexing can record multilevel data without the crosstalk noise, unlike traditional holographic storage systems. Therefore, the proposed method can utilize the full potential of the recording medium and contribute to achieving large storage capacity.

The proposed system allows the illumination of a spatially structured optical field on a material dynamically. Moreover, it is possible to investigate the optical path difference distribution of a material from the phase measurement. Owing to the above capability, the proposed system would be useful for investigating the interaction between light and polymers, and stimulating the development of applications of photo-responsive polymers.
