# Erratum: Correlation-Based Multiplexing of Complex Amplitude Data Pages in a Holographic Storage System Using Digital Holographic Techniques. *Polymers*, 2017, *9*, 375

**DOI:** 10.3390/polym9100477

**Published:** 2017-09-29

**Authors:** Teruyoshi Nobukawa, Takanori Nomura

**Affiliations:** 1 Graduate School of Systems Engineering, Wakayama University, 930 Sakaedani, Wakayama 640-8510, Japan; 2 Faculty of Systems Engineering, Wakayama University, 930 Sakaedani, Wakayama 640-8510, Japan; nom@sys.wakayama-u.ac.jp

The authors wish to make a change to the published paper [1]. In the affiliations, the institute “National Institute of Advanced Industrial Science and Technology (AIST), Electronics and Photonics Research Institute, 1-1-1 Higashi, Tsukuba, Ibaraki 305-8565, Japan” has been removed. Although Teruyoshi Nobukawa was employed by the National Institute of Advanced Technology when the manuscript was submitted, all the research described in the article has been done at the Wakayama University without any contribution from the institute. To avoid misunderstanding, we would like to remove the affiliation from the article. The authors apologize for any inconvenience caused.

The change does not affect the scientific results. The manuscript will be updated and the original will remain online on the article webpage http://www.mdpi.com/2073-4360/9/8/375.
